# Crystal structure of tetra­kis­(μ_3_-2-{[1,1-bis­(hy­droxy­meth­yl)-2-oxidoeth­yl]imino­meth­yl}phenolato)tetra­copper(II) ethanol monosolvate 2.5-hydrate

**DOI:** 10.1107/S2056989015007513

**Published:** 2015-04-22

**Authors:** Weilun Wang, Jingwen Ran

**Affiliations:** a College of Chemical Engineering, Huanggang Normal University and Hubei Key Laboratory for Processing and Application of Catalytic Materials, Huanggang 438000, People’s Republic of China

**Keywords:** crystal structure, Schiff base ligand, monoclinic tetra­nuclear copper(II) complex

## Abstract

The title compound, [Cu_4_(C_11_H_13_NO_4_)_4_]·CH_3_CH_2_OH·2.5H_2_O, is an electronically neutral tetra­nuclear copper(II) complex with a cubane-like Cu_4_O_4_ core. The complete molecule has point group symmetry 2. The phenol hy­droxy group and one of the three alcohol hy­droxy groups of each 2-{[tris­(hy­droxy­meth­yl)meth­yl]imino­meth­yl}phenol ligand are depro­ton­ated, while the secondary amine and the other two hy­droxy groups remain unchanged. The Cu^II^ atoms in the Cu_4_O_4_ core are connected by four μ_3_-O atoms from the deprotonated alcohol hy­droxy groups. Each of the penta­coordinated Cu^II^ ions has an NO_4_ distorted square-pyramidal environment through coordination to the tridentate Schiff base ligands. The Cu—N/O bond lengths span the range 1.902 (4)–1.955 (4) Å, similar to values reported for related structures. There are O—H⋯O hydrogen-bond inter­actions between the complex molecules and the ethanol and water solvent molecules, leading to the formation of a three-dimensional network. The ethanol solvent molecule is disordered about a twofold rotation axis. One of the two independent water molecules is also located on this twofold rotation axis and shows half-occupancy.

## Related literature   

For a related structure, see: Dong *et al.* (2007 ▸). For the synthesis of the 2-{[tris­(hy­droxy­meth­yl)meth­yl]imino­meth­yl}phenol ligand, see: Chumakov *et al.* (2000 ▸). 
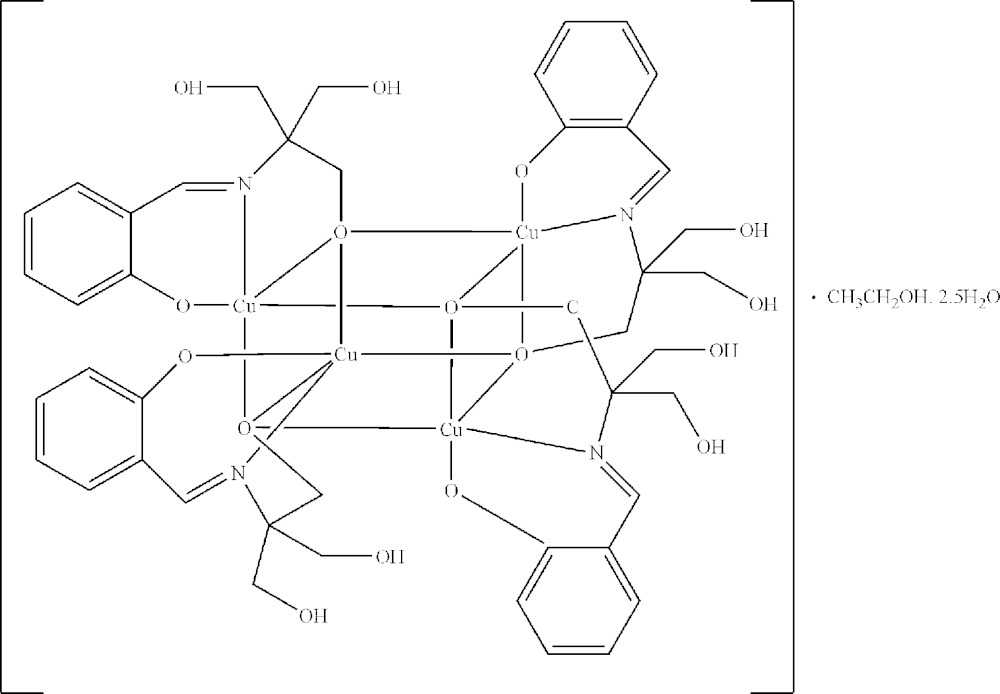


## Experimental   

### Crystal data  

[Cu_4_(C_11_H_13_NO_4_)_4_]·C_2_H_6_O·2.5H_2_O
*M* *_r_* = 1238.16
Monoclinic, 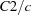

*a* = 24.651 (8) Å
*b* = 16.395 (5) Å
*c* = 18.423 (6) Å
β = 129.584 (3)°
*V* = 5738 (3) Å^3^
*Z* = 4
Mo *K*α radiation
μ = 1.53 mm^−1^
*T* = 293 K
0.38 × 0.15 × 0.14 mm

### Data collection  

Siemens SMART CCD area-detector diffractometer
Absorption correction: multi-scan (*SADABS*; Siemens, 1994 ▸) *T*_min_ = 0.594, *T*_max_ = 0.814
16163 measured reflections
5872 independent reflections
3880 reflections with *I* > 2σ(*I*)
*R*_int_ = 0.071

### Refinement  

*R*[*F* ^2^ > 2σ(*F* ^2^)] = 0.058
*wR*(*F* ^2^) = 0.188
*S* = 1.26
5872 reflections
366 parameters
96 restraints
H-atom parameters constrained
Δρ_max_ = 1.39 e Å^−3^
Δρ_min_ = −0.52 e Å^−3^

### 

Data collection: *SMART* (Siemens, 1994 ▸); cell refinement: *SMART*; data reduction: *SAINT* (Siemens, 1994 ▸); program(s) used to solve structure: *SHELXS97* (Sheldrick 2008 ▸); program(s) used to refine structure: *SHELXL2013* (Sheldrick, 2015 ▸); molecular graphics: *SHELXTL* (Siemens, 1994 ▸); software used to prepare material for publication: *SHELXTL*.

## Supplementary Material

Crystal structure: contains datablock(s) I, New_Global_Publ_Block. DOI: 10.1107/S2056989015007513/bg2551sup1.cif

Structure factors: contains datablock(s) I. DOI: 10.1107/S2056989015007513/bg2551Isup2.hkl

**Fig. 1.**
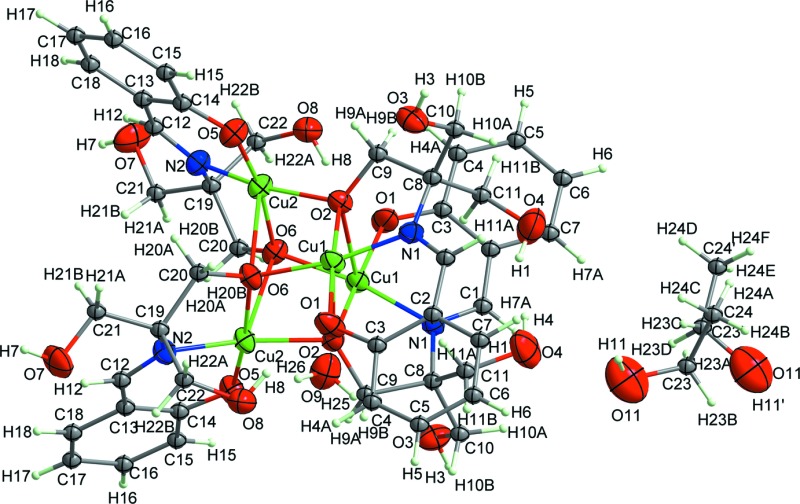
The molecular structure of the title compound. Displacement ellipsoids are drawn at the 50% probability level. Symmetry codes: #1 - x, y,-z + 1/2.

**Fig. 2.**
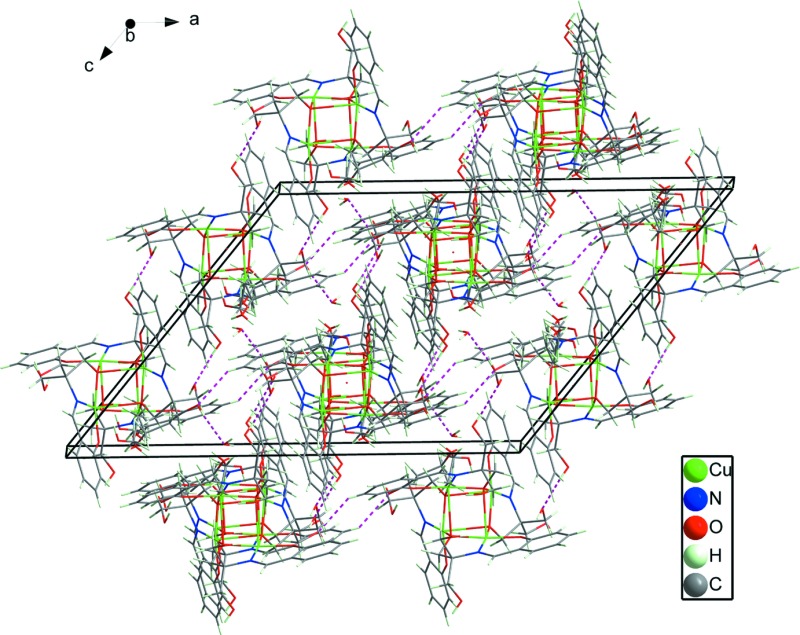
The packing diagram for the title compound, viewed down the b axis, with hydrogen bonds drawn as dashed lines.

Click here for additional data file.x y z . DOI: 10.1107/S2056989015007513/bg2551fig1.tif
The mol­ecular structure of the title compound. Displacement ellipsoids are drawn at the 50% probability level. Symmetry codes: #1 − *x*, *y*,-*z* + 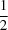.

b . DOI: 10.1107/S2056989015007513/bg2551fig2.tif
The packing diagram for the title compound, viewed down the *b* axis, with hydrogen bonds drawn as dashed lines.

CCDC reference: 628113

Additional supporting information:  crystallographic information; 3D view; checkCIF report

## Figures and Tables

**Table 1:** Hydrogen-bond geometry (, )

*D*H*A*	*D*H	H*A*	*D* *A*	*D*H*A*
O3H3O8^i^	0.82	1.94	2.706(6)	156
O7H7O9^ii^	0.82	1.98	2.769(6)	162
O8H8O1^iii^	0.82	1.83	2.641(6)	168
O9H25O3	0.82	2.15	2.925(6)	159
O9H26O5	0.82	2.09	2.824(6)	148
C12H12O7	0.93	2.31	3.011(7)	132

Symmetry codes: (i) 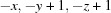; (ii) 
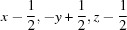
; (iii) 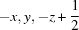.

## supplementary crystallographic information

### S1. Synthesis and crystallization

Tri­hydroxy­methyl­amino­methane (2 mmol, 242 mg), NaOH (2mmol, 80mg) and salicyl­aldehyde (2mmol, 244mg) were dissolved in ethanol (30 ml). The mixture was stirred at 333 K for 1 hour to give a yellow solution. The solution was cooled to room temperature, and solution of Cu (ClO_4_)_2_·6H_2_O (2 mmol, 741 mg) and H_2_O (20ml) were added with stirring. The mixture was stirred for 12 h at room temperature. The resulting black-green solution was filtered and allowed to stand in air for 3 d, and blue block crystals were formed at the bottom of the vessel on slow evaporation of the solvent. Yield: 31.2%. Anal. Calcd. for C_46_H_62_Cu_4_N_4_O_19_: C 44.95, H 5.08, N 4.56. Found: C 45.21, H 4.87, and N 4.59. Selected IR data (cm^-1^): 3448.02(vs.), 2917.1(w), 1625.15(vs.), 1447.05(m), 1300.17(m), 1030.55(m), 769.31(s).

### S2. Refinement details

H atoms attached to C were positioned geometrically and refined as riding atoms, with C—H = 0.97Å, and with U_iso_(H) = 1.2U_eq_(C,N). H atoms attached to O were located from a difference Fourier map and further idealized with O—H = 0.86Å and U_iso_ (H) = 1.5U_eq_ (O). The structure is completed by an ethanol solvate, disordered around a two-fold axis, and a depleted water molecule (occupation = 0.25 ) siting on the same symmetry element.

### Crystal data

**Table:**

[Cu_4_(C_11_H_13_NO_4_)_4_]·C_2_H_6_O·2.5H_2_O	*F*(000) = 2552
*M**_r_* = 1238.16	*D*_x_ = 1.433 Mg m^−^^3^
Monoclinic, *C*2/*c*	Mo *K*α radiation, λ = 0.71073 Å
Hall symbol: -C 2yc	Cell parameters from 4536 reflections
*a* = 24.651 (8) Å	θ = 2.2–26.5°
*b* = 16.395 (5) Å	µ = 1.53 mm^−^^1^
*c* = 18.423 (6) Å	*T* = 293 K
β = 129.584 (3)°	Block, blue
*V* = 5738 (3) Å^3^	0.38 × 0.15 × 0.14 mm
*Z* = 4	

### Data collection

**Table:**

Siemens SMART CCD area-detector diffractometer	5872 independent reflections
Radiation source: fine-focus sealed tube	3880 reflections with *I* > 2σ(*I*)
Graphite monochromator	*R*_int_ = 0.071
phi and ω scans	θ_max_ = 26.4°, θ_min_ = 1.6°
Absorption correction: multi-scan (*SADABS*; Siemens, 1994)	*h* = −30→30
*T*_min_ = 0.594, *T*_max_ = 0.814	*k* = −20→20
16163 measured reflections	*l* = −18→23

### Refinement

**Table:**

Refinement on *F*^2^	96 restraints
Least-squares matrix: full	Hydrogen site location: mixed
*R*[*F*^2^ > 2σ(*F*^2^)] = 0.058	H-atom parameters constrained
*wR*(*F*^2^) = 0.188	*w* = 1/[σ^2^(*F*_o_^2^) + (0.0727*P*)^2^ + 11.6375*P*] where *P* = (*F*_o_^2^ + 2*F*_c_^2^)/3
*S* = 1.25	(Δ/σ)_max_ < 0.001
5872 reflections	Δρ_max_ = 1.39 e Å^−^^3^
366 parameters	Δρ_min_ = −0.52 e Å^−^^3^

### Special details

**Table:**

Geometry. All esds (except the esd in the dihedral angle between two l.s. planes) are estimated using the full covariance matrix. The cell esds are taken into account individually in the estimation of esds in distances, angles and torsion angles; correlations between esds in cell parameters are only used when they are defined by crystal symmetry. An approximate (isotropic) treatment of cell esds is used for estimating esds involving l.s. planes.

### Fractional atomic coordinates and isotropic or equivalent isotropic displacement parameters (Å^2^)

**Table:**

	*x*	*y*	*z*	*U*_iso_*/*U*_eq_	Occ. (<1)
Cu1	0.08791 (3)	0.48191 (4)	0.34803 (5)	0.0293 (2)	
Cu2	−0.01820 (3)	0.35451 (4)	0.32582 (4)	0.0285 (2)	
N1	0.0942 (2)	0.5886 (3)	0.3980 (3)	0.0283 (10)	
N2	−0.0725 (2)	0.2569 (3)	0.2943 (3)	0.0311 (10)	
O1	0.1630 (2)	0.4990 (2)	0.3454 (3)	0.0421 (10)	
O2	0.00633 (18)	0.4701 (2)	0.3401 (2)	0.0274 (8)	
O3	0.1445 (2)	0.5896 (3)	0.5842 (3)	0.0431 (10)	
H3	0.1475	0.5826	0.6307	0.065*	
O4	0.0068 (3)	0.7335 (3)	0.3315 (4)	0.0668 (14)	
H4	0.0380	0.7263	0.3279	0.100*	
O5	0.05125 (19)	0.3281 (2)	0.4566 (3)	0.0352 (9)	
O6	0.07677 (18)	0.3689 (2)	0.3098 (2)	0.0288 (8)	
O7	−0.2014 (2)	0.1378 (3)	0.1896 (3)	0.0559 (13)	
H7	−0.2343	0.1085	0.1490	0.084*	
O8	−0.1622 (2)	0.3852 (3)	0.2567 (3)	0.0437 (10)	
H8	−0.1685	0.4202	0.2203	0.066*	
O9	0.1703 (2)	0.4295 (3)	0.5413 (3)	0.0517 (12)	
H25	0.1637	0.4672	0.5641	0.077*	
H26	0.1480	0.3891	0.5354	0.077*	
C1	0.1359 (3)	0.6463 (3)	0.4117 (4)	0.0338 (13)	
H1	0.1373	0.6938	0.4404	0.041*	
C2	0.1799 (3)	0.6426 (4)	0.3860 (4)	0.0351 (13)	
C3	0.1902 (3)	0.5706 (4)	0.3526 (4)	0.0374 (14)	
C4	0.2337 (4)	0.5775 (4)	0.3272 (5)	0.0548 (18)	
H4A	0.2417	0.5317	0.3054	0.066*	
C5	0.2641 (4)	0.6503 (5)	0.3342 (5)	0.063 (2)	
H5	0.2927	0.6527	0.3176	0.076*	
C6	0.2532 (4)	0.7202 (5)	0.3653 (5)	0.0540 (18)	
H6	0.2735	0.7694	0.3687	0.065*	
C7	0.2118 (3)	0.7157 (4)	0.3912 (4)	0.0434 (15)	
H7A	0.2047	0.7625	0.4127	0.052*	
C8	0.0459 (3)	0.6023 (3)	0.4181 (4)	0.0295 (12)	
C9	0.0123 (3)	0.5188 (3)	0.4082 (4)	0.0302 (12)	
H9A	0.0412	0.4909	0.4684	0.036*	
H9B	−0.0339	0.5273	0.3897	0.036*	
C10	0.0834 (3)	0.6367 (3)	0.5163 (4)	0.0345 (13)	
H10A	0.0967	0.6929	0.5187	0.041*	
H10B	0.0518	0.6358	0.5309	0.041*	
C11	−0.0139 (3)	0.6587 (4)	0.3433 (4)	0.0394 (14)	
H11A	−0.0422	0.6301	0.2835	0.047*	
H11B	−0.0438	0.6693	0.3595	0.047*	
C12	−0.0555 (3)	0.1969 (4)	0.3501 (4)	0.0424 (15)	
H12	−0.0872	0.1538	0.3270	0.051*	
C13	0.0094 (3)	0.1915 (4)	0.4464 (4)	0.0431 (15)	
C14	0.0587 (3)	0.2560 (3)	0.4948 (4)	0.0321 (12)	
C15	0.1180 (3)	0.2426 (4)	0.5885 (4)	0.0451 (15)	
H15	0.1515	0.2836	0.6217	0.054*	
C16	0.1276 (4)	0.1699 (5)	0.6327 (5)	0.061 (2)	
H16	0.1673	0.1629	0.6954	0.073*	
C17	0.0800 (5)	0.1079 (5)	0.5863 (6)	0.086 (3)	
H17	0.0874	0.0588	0.6166	0.103*	
C18	0.0216 (5)	0.1191 (5)	0.4954 (5)	0.075 (3)	
H18	−0.0114	0.0773	0.4644	0.090*	
C19	−0.1419 (3)	0.2622 (3)	0.1992 (4)	0.0309 (12)	
C20	0.1297 (3)	0.3087 (4)	0.3618 (4)	0.0345 (13)	
H20A	0.1161	0.2703	0.3878	0.041*	
H20B	0.1733	0.3344	0.4140	0.041*	
C21	−0.1754 (3)	0.1801 (4)	0.1510 (4)	0.0435 (15)	
H21A	−0.2136	0.1894	0.0845	0.052*	
H21B	−0.1405	0.1465	0.1565	0.052*	
C22	−0.1909 (3)	0.3110 (4)	0.2085 (4)	0.0407 (14)	
H22A	−0.2348	0.3220	0.1460	0.049*	
H22B	−0.2018	0.2777	0.2413	0.049*	
O11	0.0841 (13)	0.8666 (13)	0.4287 (16)	0.092 (4)	0.25
H11	0.0491	0.8580	0.3740	0.137*	0.25
C23	0.1211 (18)	0.938 (2)	0.4354 (19)	0.092 (4)	0.25
H23A	0.1688	0.9237	0.4627	0.110*	0.25
H23B	0.1236	0.9779	0.4767	0.110*	0.25
C24	0.0846 (18)	0.977 (2)	0.340 (2)	0.091 (4)	0.25
H24A	0.1103	1.0241	0.3464	0.136*	0.25
H24B	0.0828	0.9380	0.2991	0.136*	0.25
H24C	0.0377	0.9921	0.3130	0.136*	0.25
O11'	0.1340 (13)	1.0138 (14)	0.4583 (16)	0.091 (4)	0.25
H11'	0.1744	1.0316	0.4929	0.137*	0.25
C23'	0.1296 (19)	0.9486 (19)	0.403 (3)	0.092 (4)	0.25
H23C	0.1450	0.8989	0.4396	0.110*	0.25
H23D	0.0804	0.9413	0.3481	0.110*	0.25
C24'	0.1686 (18)	0.957 (2)	0.370 (2)	0.091 (4)	0.25
H24D	0.1612	0.9095	0.3337	0.137*	0.25
H24E	0.1530	1.0045	0.3308	0.137*	0.25
H24F	0.2179	0.9619	0.4226	0.137*	0.25
O1W	0.0000	1.0641 (15)	0.2500	0.139 (8)*	0.5

### Atomic displacement parameters (Å^2^)

**Table:**

	*U*^11^	*U*^22^	*U*^33^	*U*^12^	*U*^13^	*U*^23^
Cu1	0.0273 (4)	0.0300 (4)	0.0381 (4)	−0.0048 (3)	0.0244 (3)	−0.0062 (3)
Cu2	0.0228 (3)	0.0298 (4)	0.0287 (4)	−0.0038 (3)	0.0144 (3)	0.0006 (3)
N1	0.028 (2)	0.030 (2)	0.031 (2)	−0.004 (2)	0.021 (2)	−0.0026 (19)
N2	0.027 (2)	0.030 (2)	0.032 (3)	−0.002 (2)	0.017 (2)	0.000 (2)
O1	0.041 (2)	0.044 (2)	0.061 (3)	−0.0134 (19)	0.042 (2)	−0.017 (2)
O2	0.0236 (18)	0.0312 (19)	0.029 (2)	−0.0046 (16)	0.0173 (16)	−0.0057 (15)
O3	0.039 (2)	0.056 (3)	0.029 (2)	0.008 (2)	0.0191 (19)	0.0007 (19)
O4	0.075 (4)	0.046 (3)	0.095 (4)	0.013 (3)	0.062 (3)	0.020 (3)
O5	0.026 (2)	0.037 (2)	0.031 (2)	−0.0034 (17)	0.0135 (17)	0.0042 (17)
O6	0.0212 (18)	0.0306 (19)	0.030 (2)	0.0035 (15)	0.0143 (16)	−0.0021 (16)
O7	0.052 (3)	0.063 (3)	0.048 (3)	−0.033 (2)	0.030 (2)	−0.011 (2)
O8	0.057 (3)	0.041 (2)	0.045 (3)	0.002 (2)	0.038 (2)	0.002 (2)
O9	0.037 (2)	0.047 (3)	0.065 (3)	0.005 (2)	0.029 (2)	−0.001 (2)
C1	0.033 (3)	0.033 (3)	0.030 (3)	−0.007 (2)	0.018 (3)	−0.007 (2)
C2	0.029 (3)	0.043 (3)	0.031 (3)	−0.011 (3)	0.018 (3)	−0.005 (3)
C3	0.029 (3)	0.049 (4)	0.039 (3)	−0.010 (3)	0.024 (3)	−0.005 (3)
C4	0.057 (4)	0.062 (4)	0.073 (5)	−0.022 (4)	0.054 (4)	−0.021 (4)
C5	0.066 (5)	0.083 (6)	0.069 (5)	−0.026 (4)	0.056 (5)	−0.013 (4)
C6	0.045 (4)	0.066 (5)	0.048 (4)	−0.026 (4)	0.028 (3)	−0.003 (4)
C7	0.047 (4)	0.043 (3)	0.042 (4)	−0.018 (3)	0.029 (3)	−0.008 (3)
C8	0.031 (3)	0.033 (3)	0.029 (3)	0.002 (2)	0.022 (3)	−0.001 (2)
C9	0.027 (3)	0.033 (3)	0.035 (3)	0.000 (2)	0.022 (3)	−0.003 (2)
C10	0.035 (3)	0.038 (3)	0.035 (3)	0.000 (3)	0.024 (3)	−0.005 (3)
C11	0.038 (3)	0.046 (3)	0.040 (3)	0.007 (3)	0.027 (3)	−0.001 (3)
C12	0.038 (3)	0.039 (3)	0.041 (3)	−0.014 (3)	0.021 (3)	0.000 (3)
C13	0.038 (3)	0.041 (3)	0.036 (3)	−0.003 (3)	0.017 (3)	0.005 (3)
C14	0.026 (3)	0.040 (3)	0.030 (3)	0.004 (2)	0.017 (3)	0.002 (2)
C15	0.036 (3)	0.050 (4)	0.037 (3)	0.000 (3)	0.018 (3)	0.007 (3)
C16	0.051 (4)	0.066 (5)	0.039 (4)	0.006 (4)	0.016 (3)	0.018 (4)
C17	0.084 (6)	0.056 (5)	0.056 (5)	−0.015 (5)	0.016 (5)	0.023 (4)
C18	0.076 (6)	0.048 (4)	0.056 (5)	−0.020 (4)	0.020 (4)	0.013 (4)
C19	0.024 (3)	0.037 (3)	0.030 (3)	−0.006 (2)	0.016 (2)	−0.002 (2)
C20	0.030 (3)	0.039 (3)	0.035 (3)	0.007 (3)	0.021 (3)	0.003 (3)
C21	0.039 (3)	0.045 (3)	0.041 (4)	−0.020 (3)	0.023 (3)	−0.010 (3)
C22	0.032 (3)	0.051 (4)	0.042 (3)	−0.002 (3)	0.025 (3)	0.002 (3)
O11	0.072 (6)	0.080 (6)	0.075 (6)	0.012 (5)	0.025 (5)	0.021 (5)
C23	0.072 (6)	0.080 (6)	0.075 (6)	0.012 (5)	0.025 (5)	0.021 (5)
C24	0.072 (6)	0.080 (6)	0.075 (6)	0.012 (5)	0.026 (5)	0.021 (5)
O11'	0.073 (6)	0.080 (6)	0.075 (6)	0.012 (5)	0.026 (5)	0.021 (5)
C23'	0.072 (6)	0.080 (6)	0.075 (6)	0.012 (5)	0.025 (5)	0.021 (5)
C24'	0.072 (6)	0.080 (6)	0.075 (6)	0.012 (5)	0.026 (5)	0.021 (5)

### Geometric parameters (Å, º)

**Table:**

Cu1—O1	1.902 (4)	C9—H9B	0.9700
Cu1—O2	1.931 (4)	C10—H10A	0.9700
Cu1—N1	1.936 (4)	C10—H10B	0.9700
Cu1—O6	1.939 (3)	C11—H11A	0.9700
Cu2—O5	1.915 (4)	C11—H11B	0.9700
Cu2—N2	1.924 (4)	C12—C13	1.448 (8)
Cu2—O6^i^	1.945 (4)	C12—H12	0.9300
Cu2—O2	1.955 (4)	C13—C18	1.402 (9)
N1—C1	1.298 (7)	C13—C14	1.417 (8)
N1—C8	1.473 (6)	C14—C15	1.395 (8)
N2—C12	1.285 (7)	C15—C16	1.376 (9)
N2—C19	1.478 (7)	C15—H15	0.9300
O1—C3	1.317 (7)	C16—C17	1.365 (10)
O2—C9	1.413 (6)	C16—H16	0.9300
O3—C10	1.425 (7)	C17—C18	1.356 (10)
O3—H3	0.8200	C17—H17	0.9300
O4—C11	1.398 (7)	C18—H18	0.9300
O4—H4	0.8200	C19—C21	1.532 (8)
O5—C14	1.328 (6)	C19—C20^i^	1.540 (7)
O6—C20	1.414 (6)	C19—C22	1.546 (8)
O6—Cu2^i^	1.945 (4)	C20—C19^i^	1.540 (7)
O7—C21	1.408 (7)	C20—H20A	0.9700
O7—H7	0.8200	C20—H20B	0.9700
O8—C22	1.403 (7)	C21—H21A	0.9700
O8—H8	0.8200	C21—H21B	0.9700
O9—H25	0.8200	C22—H22A	0.9700
O9—H26	0.8215	C22—H22B	0.9700
C1—C2	1.436 (8)	O11—C23	1.446 (19)
C1—H1	0.9300	O11—H11	0.8200
C2—C7	1.402 (8)	C23—C24	1.512 (18)
C2—C3	1.427 (8)	C23—H23A	0.9700
C3—C4	1.422 (8)	C23—H23B	0.9700
C4—C5	1.371 (10)	C24—H24A	0.9600
C4—H4A	0.9300	C24—H24B	0.9600
C5—C6	1.383 (10)	C24—H24C	0.9600
C5—H5	0.9300	O11'—C23'	1.437 (19)
C6—C7	1.376 (9)	O11'—H11'	0.8200
C6—H6	0.9300	C23'—C24'	1.433 (19)
C7—H7A	0.9300	C23'—H23C	0.9700
C8—C10	1.522 (7)	C23'—H23D	0.9700
C8—C11	1.528 (8)	C24'—H24D	0.9600
C8—C9	1.549 (7)	C24'—H24E	0.9600
C9—H9A	0.9700	C24'—H24F	0.9600
			
O1—Cu1—O2	174.79 (17)	C8—C11—H11B	108.4
O1—Cu1—N1	95.44 (17)	H11A—C11—H11B	107.4
O2—Cu1—N1	83.71 (16)	N2—C12—C13	124.7 (5)
O1—Cu1—O6	92.64 (16)	N2—C12—H12	117.7
O2—Cu1—O6	88.70 (14)	C13—C12—H12	117.7
N1—Cu1—O6	170.43 (17)	C18—C13—C14	118.7 (6)
O5—Cu2—N2	94.79 (17)	C18—C13—C12	117.1 (6)
O5—Cu2—O6^i^	168.55 (16)	C14—C13—C12	124.1 (5)
N2—Cu2—O6^i^	84.78 (16)	O5—C14—C15	118.5 (5)
O5—Cu2—O2	95.65 (15)	O5—C14—C13	123.9 (5)
N2—Cu2—O2	160.56 (17)	C15—C14—C13	117.6 (5)
O6^i^—Cu2—O2	88.17 (14)	C16—C15—C14	121.2 (6)
C1—N1—C8	120.5 (5)	C16—C15—H15	119.4
C1—N1—Cu1	124.4 (4)	C14—C15—H15	119.4
C8—N1—Cu1	115.2 (3)	C17—C16—C15	121.3 (7)
C12—N2—C19	122.3 (5)	C17—C16—H16	119.3
C12—N2—Cu2	125.9 (4)	C15—C16—H16	119.3
C19—N2—Cu2	111.5 (3)	C18—C17—C16	119.0 (7)
C3—O1—Cu1	124.8 (4)	C18—C17—H17	120.5
C9—O2—Cu1	111.2 (3)	C16—C17—H17	120.5
C9—O2—Cu2	121.0 (3)	C17—C18—C13	122.2 (7)
Cu1—O2—Cu2	108.99 (17)	C17—C18—H18	118.9
C10—O3—H3	109.5	C13—C18—H18	118.9
C11—O4—H4	109.9	N2—C19—C21	115.1 (5)
C14—O5—Cu2	125.4 (3)	N2—C19—C20^i^	106.1 (4)
C20—O6—Cu1	124.4 (3)	C21—C19—C20^i^	107.1 (4)
C20—O6—Cu2^i^	113.3 (3)	N2—C19—C22	107.2 (4)
Cu1—O6—Cu2^i^	113.58 (17)	C21—C19—C22	110.5 (5)
C21—O7—H7	109.3	C20^i^—C19—C22	110.8 (5)
C22—O8—H8	109.5	O6—C20—C19^i^	112.0 (4)
H25—O9—H26	110.0	O6—C20—H20A	109.2
N1—C1—C2	125.2 (5)	C19^i^—C20—H20A	109.2
N1—C1—H1	117.4	O6—C20—H20B	109.2
C2—C1—H1	117.4	C19^i^—C20—H20B	109.2
C7—C2—C3	119.4 (5)	H20A—C20—H20B	107.9
C7—C2—C1	116.8 (5)	O7—C21—C19	113.0 (5)
C3—C2—C1	123.8 (5)	O7—C21—H21A	109.0
O1—C3—C4	118.8 (5)	C19—C21—H21A	109.0
O1—C3—C2	124.1 (5)	O7—C21—H21B	109.0
C4—C3—C2	117.1 (5)	C19—C21—H21B	109.0
C5—C4—C3	121.3 (6)	H21A—C21—H21B	107.8
C5—C4—H4A	119.4	O8—C22—C19	113.8 (4)
C3—C4—H4A	119.4	O8—C22—H22A	108.8
C4—C5—C6	121.4 (6)	C19—C22—H22A	108.8
C4—C5—H5	119.3	O8—C22—H22B	108.8
C6—C5—H5	119.3	C19—C22—H22B	108.8
C7—C6—C5	118.9 (6)	H22A—C22—H22B	107.7
C7—C6—H6	120.6	C23—O11—H11	110.8
C5—C6—H6	120.6	O11—C23—C24	111.4 (18)
C6—C7—C2	122.0 (6)	O11—C23—H23A	109.4
C6—C7—H7A	119.0	C24—C23—H23A	109.4
C2—C7—H7A	119.0	O11—C23—H23B	109.4
N1—C8—C10	112.3 (4)	C24—C23—H23B	109.4
N1—C8—C11	109.6 (4)	H23A—C23—H23B	108.0
C10—C8—C11	110.8 (4)	C23—C24—H24A	109.5
N1—C8—C9	107.0 (4)	C23—C24—H24B	109.5
C10—C8—C9	110.0 (4)	H24A—C24—H24B	109.5
C11—C8—C9	107.0 (4)	C23—C24—H24C	109.5
O2—C9—C8	110.5 (4)	H24A—C24—H24C	109.5
O2—C9—H9A	109.6	H24B—C24—H24C	109.5
C8—C9—H9A	109.6	C23'—O11'—H11'	108.8
O2—C9—H9B	109.6	C24'—C23'—O11'	118 (2)
C8—C9—H9B	109.6	C24'—C23'—H23C	107.9
H9A—C9—H9B	108.1	O11'—C23'—H23C	107.9
O3—C10—C8	110.1 (4)	C24'—C23'—H23D	107.9
O3—C10—H10A	109.6	O11'—C23'—H23D	107.9
C8—C10—H10A	109.6	H23C—C23'—H23D	107.2
O3—C10—H10B	109.6	C23'—C24'—H24D	109.5
C8—C10—H10B	109.6	C23'—C24'—H24E	109.5
H10A—C10—H10B	108.2	H24D—C24'—H24E	109.5
O4—C11—C8	115.6 (5)	C23'—C24'—H24F	109.5
O4—C11—H11A	108.4	H24D—C24'—H24F	109.5
C8—C11—H11A	108.4	H24E—C24'—H24F	109.5
O4—C11—H11B	108.4		

Symmetry code: (i) −*x*, *y*, −*z*+1/2.

### Hydrogen-bond geometry (Å, º)

**Table:**

*D*—H···*A*	*D*—H	H···*A*	*D*···*A*	*D*—H···*A*
O3—H3···O8^ii^	0.82	1.94	2.706 (6)	156
O7—H7···O9^iii^	0.82	1.98	2.769 (6)	162
O8—H8···O1^i^	0.82	1.83	2.641 (6)	168
O9—H25···O3	0.82	2.15	2.925 (6)	159
O9—H26···O5	0.82	2.09	2.824 (6)	148
C12—H12···O7	0.93	2.31	3.011 (7)	132

Symmetry codes: (i) −*x*, *y*, −*z*+1/2; (ii) −*x*, −*y*+1, −*z*+1; (iii) *x*−1/2, −*y*+1/2, *z*−1/2.
