# Supplementary figures and images for: Crystal structure of tetra­kis­(μ3-2-{[1,1-bis­(hy­droxy­meth­yl)-2-oxidoeth­yl]imino­meth­yl}phenolato)tetra­copper(II) ethanol monosolvate 2.5-hydrate
Source: Acta Crystallogr E Crystallogr Commun. 2015 Apr 22;71(Pt 5):m116–7. doi: 10.1107/S2056989015007513 (PMC4420116; doi:10.1107/S2056989015007513)

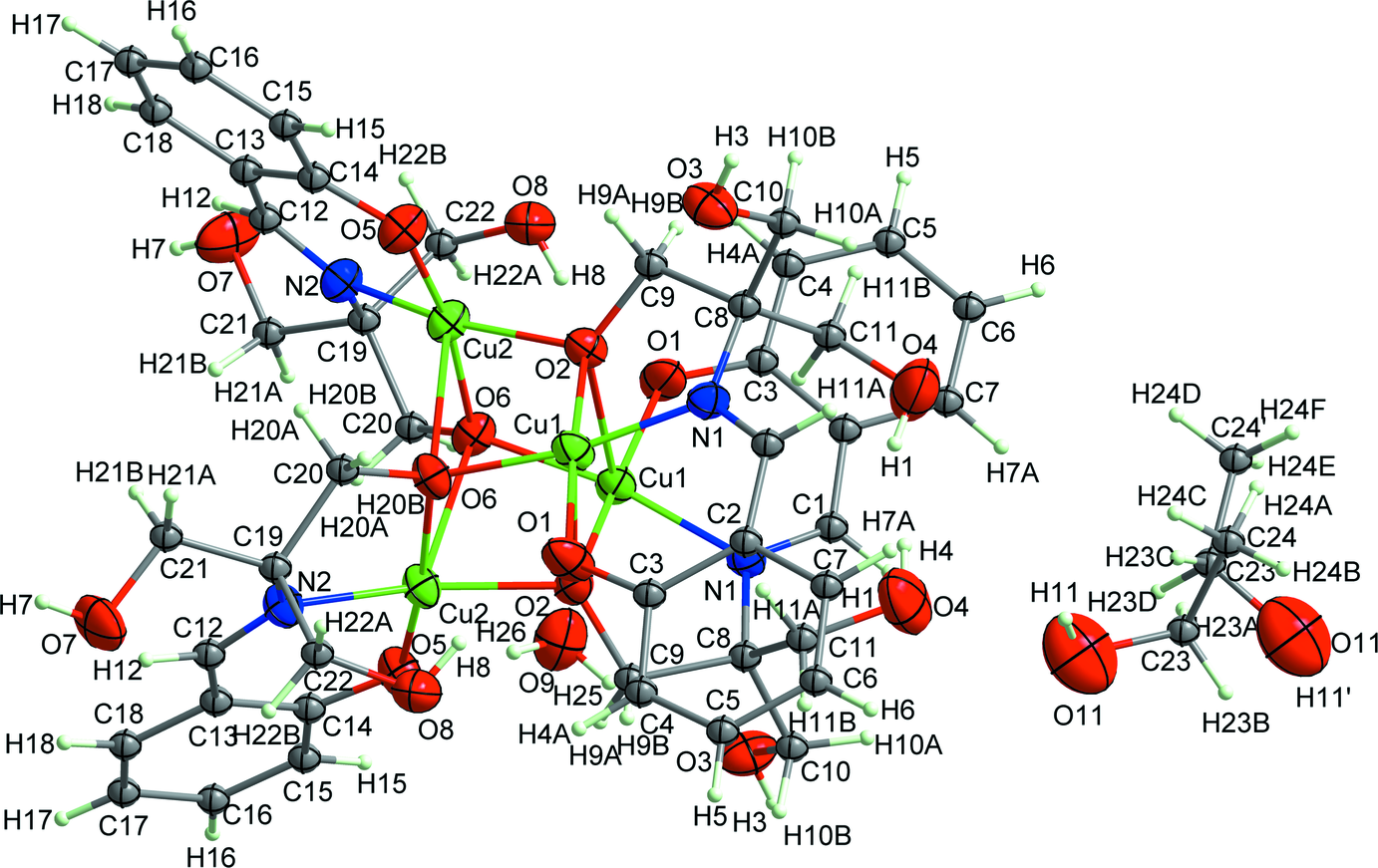

Supplement: Supplementary file 3 [file e-71-0m116-fig1.tif]

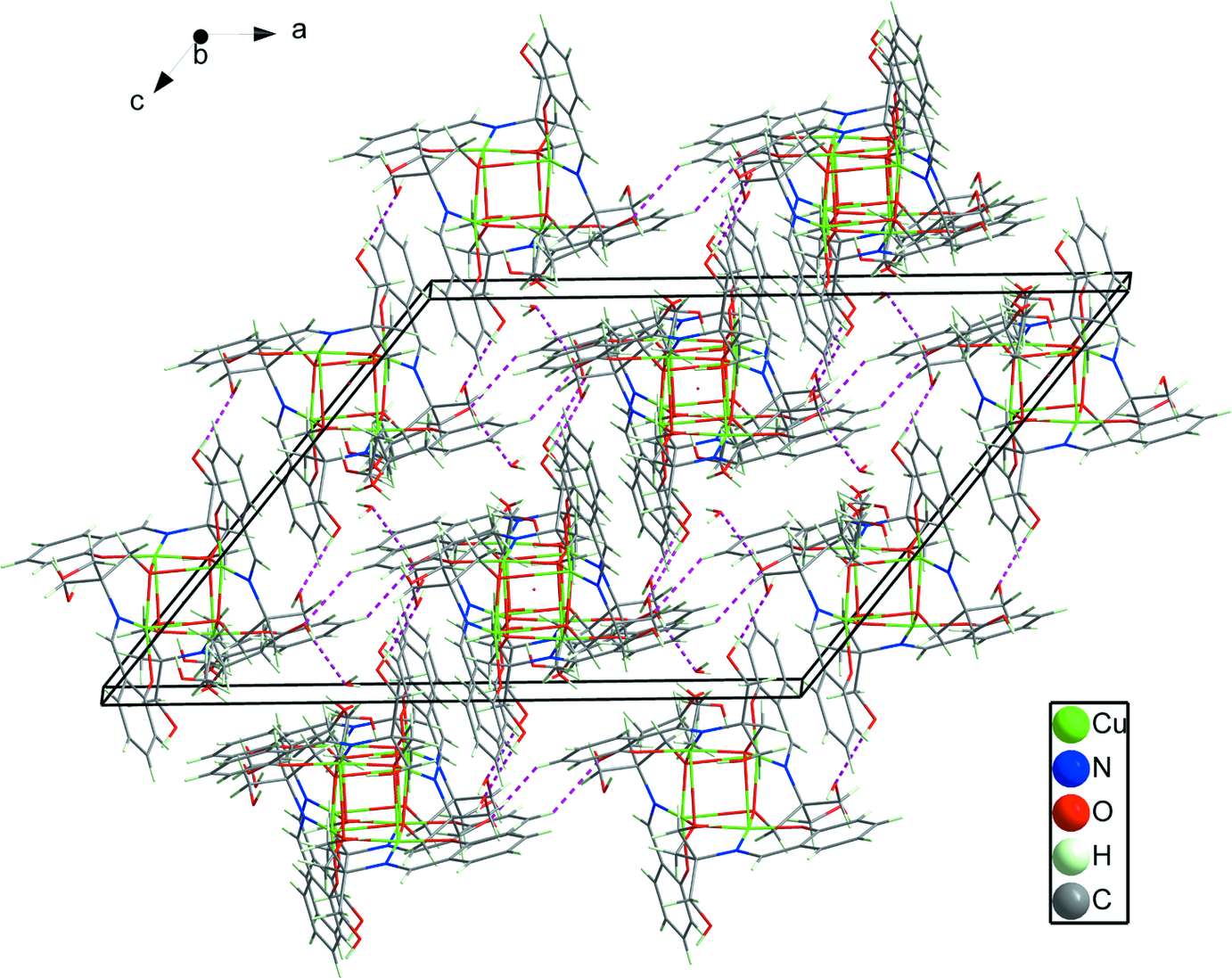

Supplement: Supplementary file 4 [file e-71-0m116-fig2.tif]
